# An Update on Anti-COVID-19 Vaccines and the Challenges to Protect Against New SARS-CoV-2 Variants

**DOI:** 10.3390/pathogens14010023

**Published:** 2025-01-01

**Authors:** Fábio Mambelli, Ana Carolina V. S. C. de Araujo, Jéssica P. Farias, Kivia Q. de Andrade, Luis C. S. Ferreira, Paola Minoprio, Luciana C. C. Leite, Sergio C. Oliveira

**Affiliations:** 1 Departamento de Imunologia, Instituto de Ciências Biomédicas, Universidade de São Paulo, São Paulo 05508-000, Brazil; fabio_mambelli@yahoo.com.br (F.M.); carolvalente97@gmail.com (A.C.V.S.C.d.A.); kiviaqandrade@gmail.com (K.Q.d.A.); 2 Institut Pasteur de São Paulo, São Paulo 05508-020, Brazil; lcsf@usp.br (L.C.S.F.); paola.minoprio@pasteur.fr (P.M.); 3 Departamento de Microbiologia, Instituto de Ciências Biomédicas, Universidade de São Paulo, São Paulo 05508-000, Brazil; jessicapires435@usp.br; 4 Laboratório de Desenvolvimento de Vacinas, Instituto Butantan, São Paulo 05503-900, Brazil; luciana.leite@butantan.gov.br

**Keywords:** COVID-19, SARS-CoV-2, vaccine, variants, mucosal, BCG

## Abstract

The COVID-19 pandemic has posed a significant threat to global health systems, with extensive impacts across many sectors of society. The pandemic has been responsible for millions of deaths worldwide since its first identification in late 2019. Several actions have been taken to prevent the disease, including the unprecedented fast development and global vaccination campaigns, which were pivotal in reducing symptoms and deaths. Given the impact of the pandemic, the continuous changes of the virus, and present vaccine technologies, this review analyzes how, so far, we have met the challenge posed by the emergence of new variants and discusses how next-generation pan-coronavirus vaccines, with enhanced longevity and breadth of immune responses, may be tackled with alternative administration routes and antigen delivery platforms. By addressing these critical aspects, this review aims to contribute to the ongoing efforts to achieve long-term control of COVID-19, stimulating the discussion and work on next-generation vaccines capable of facing future waves of infection.

## 1. Introduction

The Coronavirus Disease 2019 (COVID-19) pandemic has posed a significant threat to global health systems, with extensive impacts across many areas of society. Since its outbreak in late 2019 in Wuhan, China, it has been responsible for millions of deaths worldwide. As a result of its elevated transmissibility, the Severe Acute Respiratory Syndrome Coronavirus 2 (SARS-CoV-2) quickly spread across several countries, overwhelming healthcare services and establishing a global pandemic, as designated by the World Health Organization (WHO) in March 2020. Several efforts were required to manage this critical situation, which included genomic surveillance programs, lockdown countermeasures, and heavy investments in the development of diagnostics, therapies, and vaccines [1,2]. Overall, the joint efforts from the international scientific community led to a growing understanding of the virus’ pathogenesis, culminating in the development of hundreds of vaccine candidates at an unprecedented pace. Vaccines were developed on several technological platforms, including inactivated microorganisms, nucleic acid, peptides, recombinant proteins, VLPs, and others. Consequently, regulatory agencies issued emergency use approvals for protective and safe vaccines in late 2020, which was paramount for slowing the pandemic pace. Millions of vaccine doses (manufactured by Pfizer-BioNTech, Moderna, Sinovac Biotech, Oxford-AstraZeneca, and Johnson & Johnson’s Janssen, among others) were made available all over the world together with vaccination campaigns [3]. Although there was success in reducing viral spread, disease severity, hospitalizations, and deaths, mutated strains of SARS-CoV-2 soon became a major concern, jeopardizing both natural and vaccine-induced immunity [4,5,6].

Several mutations in viral Spike protein were largely responsible for the evasion of neutralizing antibodies, thus affecting vaccine efficacy and increasing the transmissibility of these emerging Variants of Concern (VOCs), leading to new waves of infection. Due to the growing list of VOCs (including Alpha, Beta, Gamma, Delta, and Omicron sublineages) and the waning immunity induced by the available immunizers, updated vaccine formulations were required to maintain protective immunity in the population [7]. Despite the WHO declaring the end of the Public Health Emergency of International Concern status of COVID-19 in March 2023, the spread of SARS-CoV-2 VOCs is still being observed [6]. Therefore, ongoing surveillance and investment in the development of formulations aiming to induce broader and more durable immunity against emerging variants is still an urgent need.

Given the significant impact of SARS-CoV-2 in recent history, this review analyzes the COVID-19 pandemic scenario, discusses vaccine development and efficacy against the ancestral Wuhan strain as well as VOCs, and integrates the results from relevant pre-clinical studies on novel anti-COVID-19 vaccine candidates, focusing on the implications of emerging variants and vaccine efficacy. It also examines the current landscape, offering insights into future directions for next-generation vaccines. By addressing these critical aspects, this review aims to contribute to the ongoing efforts to achieve long-term control of COVID-19.

## 2. Available Anti-COVID-19 Vaccines: Where Do We Stand?

At the beginning of the COVID-19 pandemic, significant effort was devoted to characterizing the immune response against COVID-19. Previous data on the pathophysiology learned on human coronaviruses (such as OC43, HKU1, MERS-CoV, and SARS-CoV) helped pave the initial investigations regarding SARS-CoV-2 infections. The mechanism through which the SARS-CoV-2 virus attaches to the angiotensin-converting enzyme 2 (ACE2) receptors from cells, leading to their invasion, is currently well established. Its viral genome consists of a single-stranded positive-sense RNA with approximately 29.9 kilobases in length and codes for non-structural proteins required for viral replication and pathogenesis, as well as structural proteins such as the Spike (S), Nucleocapsid (N), Envelope (E) and Membrane (M). Here, the Spike protein has been one of the main targets of general study due to its role in viral entry into host cells via the ACE2 receptor [8,9,10,11]. Once infection is established, a potent antiviral immune response via cytotoxic and helper T cells is evoked. The outcome of the infection mainly depends on the coordination between innate and adaptive immune responses in its early stages. In this context, when T cells fail to provide suitable immune responses, viral replication can occur in an exacerbated manner. Recognition of viral pathogen-associated and damage-associated molecular patterns (PAMPs and DAMPs, respectively) leads to increased secretion of inflammatory cytokines, such as IFN-γ, IL-6, CXCL10, and others, aggravating the immune scenario [12,13]. Consequently, elevated migration of neutrophils and macrophages to the inflammatory site contributes to increased tissue damage and the cytokine storm, as observed in severe cases of COVID-19 [12,13]. However, when prompt and adequate immune responses are in place, lower respiratory tract infected epithelial cells are properly phagocytosed by antigen-presenting cells (APCs), leading to sufficient CD4^+^ and CD8^+^ T cell clonal expansion. Consequently, upon antigen exposure, Th0 cells polarize towards Th1, with CD8^+^ T cells targeting infected cells and IFN-γ secretion, leading to viral elimination, as well as towards Th2, with CD4^+^ T cells stimulating B cells, leading to antibody secretion and viral neutralization [9,13]. However, differences in genetic background, immune status, comorbidities, and environmental factors might contribute to different outcomes, ranging from asymptomatic and mild cases to severe disease and death. With millions of deaths worldwide, mass vaccination was critical for changing the pandemic scenario, as it significantly reduced the risk of severe disease and death, being safer and presenting fewer side effects when compared to the unpredictable outcomes of natural infection.

**Figure 1:**
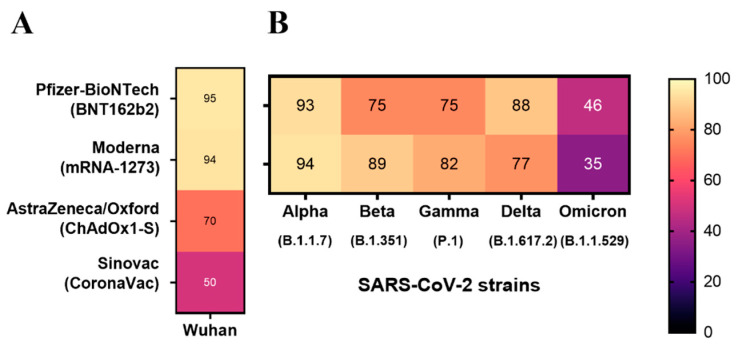
The heatmap illustrates the approximate percentage of protection against symptomatic COVID-19 with each of the four approved anti-COVID-19 vaccines from different platforms: mRNA-based vaccines such as BNT162b2 (Pfizer-BioNTech) and mRNA-1273 (Moderna), adenoviral vectors such as ChAdOx1-S (AstraZeneca/Oxford), and inactivated virus such as CoronaVac (Sinovac Biotech): (**A**) Wuhan ancestral and (**B**) Alpha (B.1.1.7), Beta (B.1.351), Gamma (P.1), Delta (B.1.617.2), and Omicron (B.1.1.529) strains. Approximate percentages were calculated as the average percentage from reports with 95% confidence interval and detected one to three months after the second dose of each vaccine. References: [9,14,16,18,19,21,23,24].

Given the above, in December 2020, the Food and Drug Administration (FDA, Silver Spring, MD, USA) issued the first emergency use authorization for the Pfizer-BioNTech COVID-19 vaccine (aka. BNT162b2) and later for Moderna (aka. mRNA-1273), both mRNA-based platforms targeting the Spike protein [14,15]. For the first time used in humans, the rationale was to deliver the mRNA Spike-coding sequence leading to translation in host cells and induction of immune response. In Brazil, later in January 2021, the Brazilian Health Regulatory Agency (Anvisa) approved the emergency use of the CoronaVac (Sinovac Biotech, Beijing, China) and ChAdOx1-S (Oxford-AstraZeneca) vaccines. CoronaVac represents the so-called conventional (first-generation) platform (inactivated whole-virus that cannot infect and replicate), while the non-replicating adenoviral vector containing the Spike coding sequence (ChAdOx1-S and others) also had a debut in the repertoire of human vaccines [14]. Despite different approaches, these anti-COVID-19 formulations (and others later approved for use and not exemplified here but reviewed elsewhere [15,16,17]) were meant to induce neutralizing antibodies (nAbs) against Spike and impair viral infection and severe forms of the disease. As displayed in Figure 1A, the mRNA-based vaccines demonstrated the highest efficacy rates in the clinical trials, with the BNT162b2 vaccine showing around 95% efficacy and mRNA-1273 approximately 94% in preventing symptomatic COVID-19 after a two-dose immunization regimen. The ChAdOx1-S and CoronaVac, two additional vaccines largely used in Brazil, displayed around 70 and 50% protection against the virus [9,18,19,20]. However, a significant decline in detected antibody levels was observed for all the available vaccines after approximately 6 months, highlighting the declining serum levels of antiviral antibodies and the need for booster doses [21,22].

In that sense, in late 2021, the FDA amended booster doses and the “mix-and-match strategy” for the mRNA vaccines, as the population would be boosted with any available vaccine. This heterologous vaccination strategy, which involves combining doses of different vaccines, has emerged as an effective approach to enhance the immune response, broadening protection against COVID-19. Studies demonstrated that combining an initial dose of a viral vector vaccine (e.g., ChAdOx1-S) with a booster dose of an mRNA vaccine (e.g., mRNA-1273) induced a stronger and longer-lasting immune response when compared to homologous immunization (e.g., ChAdOx1-S/ChAdOx1-S). Indeed, cross-sectional analyses of a Brazilian population subjected to diverse combinations of anti-COVID-19 vaccines (including ChAdOx1-S and CoronaVac) demonstrated the relevance of a second booster dose with the mRNA vaccine to improve or restore robust humoral immune status [25]. This strategy has also proven valuable in addressing vaccine shortages and mitigating the spread of upcoming variants [26,27,28].

**Figure 2:**
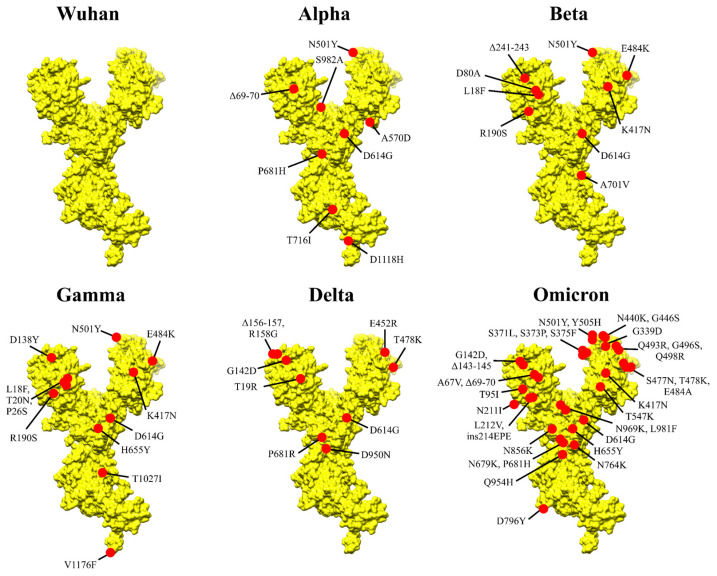
Graphical representation of the Spike protein from different SARS-CoV-2 Variants of Concern. The monomeric Spike from Wuhan ancestral strain is depicted in yellow (PDB: 6VSB), and key mutations are depicted as red dots for Alpha (B.1.1.7), Beta (B.1.351), Gamma (P.1), Delta (B.1.617.2), and Omicron (B.1.1.529). The illustration was prepared with the software Chimera 1.18 (developed by the Resource for Biocomputing, Visualization, and Informatics at the University of California, San Francisco).

The emergence of SARS-CoV-2 variants has posed significant challenges to vaccine efficacy as they spread across many countries. These variants, particularly those classified as Variants of Concern (VOCs), exhibit increased transmissibility and virulence and often evade antibody protection from previous infection or vaccination. The Alpha (B.1.1.7), Beta (B.1.351), Gamma (P.1), and Delta (B.1.617.2) variants were first identified in 2020, in the United Kingdom, South Africa, Brazil, and India, respectively, each with distinct characteristics in terms of transmissibility and immune evasion [17,29,30], caused by several mutations as illustrated in Figure 2 on the Spike protein. Alpha presented 23 different mutations in the viral genome (including 8 in the Spike region), granting higher transmissibility and moderate immune evasion. It quickly spread over many countries, leading to a sharp rise in COVID-19 cases worldwide [31]. Beta, on the other hand, exhibited nine mutations in the Spike region and diminished transmission. However, it presented higher immune escape, significantly reducing the effectiveness of nAbs from vaccines, raising concerns about vaccine efficacy. Gamma displayed a higher transmissibility rate than Beta but lesser than Alpha while still demonstrating considerate resistance to antibody neutralization. It contained 12 mutations in the Spike protein and quickly became dominant in several regions of South America, contributing to surges of reinfection in Brazil [32]. Delta, in turn, possessed significant transmissibility (twice as infectious as the previous ones), quickly becoming the widest global spread variant. It was also associated with increased severity of outcomes, including higher rates of hospitalization. Delta presented nine key mutations in Spike, contributing to moderate escape from vaccine-induced immune protection [17,29,30]. Despite the concern raised by the variants, as they presented reduced neutralization by vaccine-induced antibodies, immunization still provided substantial protection against severe outcomes (as illustrated in Figure 1B). This was especially true due to the booster dose strategy, which aided in enhancing protection over these variants, circumventing the waning immunity to some extent [33,34]. It is important to highlight the importance of adaptive cell-mediated immunity supporting humoral responses, especially in cases of reduced nAbs titers. Most individuals recovering from COVID-19 present a long-lasting specific memory T cell response. Unlike the nAbs response, which is majorly directed to RBD epitopes, T cell responses target a broader range of viral proteins [35]. Here, recognition of conserved viral epitopes by T cells (especially more conserved regions such as the nucleocapsid) plays a pivotal role [36]. Studies among oncologic patients with COVID-19 and impaired effective humoral response have shown those with higher CD8^+^ T cell counts presented elevated survival rates [37]. Also, it is known that robust CD4^+^ T cell response is critical for generating effective neutralizing antibodies during initial SARS-CoV-2 infection [38], as they stimulate and support B cell differentiation and antibody secretion. Regarding immune responses to VOCs in vaccinated individuals, SARS-CoV-2-specific T cell responses have shown durability and cross-reactivity, being detected at significant levels [38,39,40].

Concerning the Omicron (B.1.1.529; BA.1) variant, first detected in late 2021 in South Africa, it introduced further complexity to the issue of immune escape, emerging as a highly concerning variant. Due to its alarming number of mutations throughout the genome, including more than thirty mutations in the Spike coding sequence (as illustrated in Figure 2), Omicron exhibited significant differences from the Wuhan ancestral strain. With enhanced transmissibility, easily evading natural and vaccine-induced immunity (as illustrated in Figure 1B), it quickly outcompeted Delta and spread globally within weeks of its detection, leading to massive surges of COVID-19 [17,41]. Shortly after, several sub-lineages of Omicron emerged, including BA.2, BA.4, BA.5, XBB, and others, each displaying specific mutations, influencing transmissibility and immune escape [42]. Studies reported a significant decrease in neutralization by vaccine-induced antibodies when compared to the ancestral strain, leading to a rise in breakthrough infections and prompting the development of new vaccine formulations [29,30].

As an alternative to containing the spread of Omicron and its subvariants, some countries followed WHO recommendations and recommended third and fourth booster doses of monovalent vaccines in their population [43]. In Brazil, studies indicated that administration of a third dose of the mRNA vaccine (BNT162b2) reduced susceptibility to infection by the Omicron variant. Further studies demonstrated that although the administration of a fourth dose was not capable of increasing serum levels of anti-SARS-CoV-2 antibodies, it enhanced the serum concentration of neutralizing antibody response against the Omicron variants [44,45]. Despite the responses against the first Omicron subvariants, the strategy based on repeated booster doses and the continued emergence of new variants and subvariants culminate in reduced general efficacy of the first-generation COVID-19 vaccines, thus requiring the development of second-generation vaccines aiming for longer-lasting protective effects [46].

In this setting, the FDA issued the need for bivalent vaccines incorporating antigens from both the ancestral strain and prevalent variants, aiming to broaden immune coverage and circumvent immune evasion from variants. This approach was meant to induce a more versatile and effective immune response capable of neutralizing multiple viral forms, like the approach used for annual influenza vaccines. Pfizer and Moderna’s vaccines were updated and granted emergency use authorization for their bivalent formulations, mRNA vaccines coding for ancestral Spike WA1/2020 and BA.4/5 Omicron proteins. This strategy elicited strong and broader immunity, providing neutralizing activity against the variants [47,48,49]. These findings were in accordance with expectations since the bivalent vaccine formulations were administered mainly to individuals who had received two or more doses of the monovalent vaccines, resulting in higher neutralization activity against the Wuhan strain when compared to initial Omicron variants [50]. On the other hand, antibody responses against Omicron variants were primed with low concentrations of neutralizing antibodies, although sufficient to achieve statistically significant differentiation when compared to monovalent immunization vaccines [50]. The upcoming strategy for vaccine boosters in fall 2023 [51] considered removing the ancestral Spike strain and proceeding with monovalent formulations with a focus on the circulating variants [52]. At the beginning of this year, for example, Brazil’s regulatory agency (Anvisa) approved the registration of two monovalent vaccines: Zalika, manufactured by India’s Serum Institute, and Spikevax from Moderna. Both vaccines were updated to target the XBB1.5 variant [53,54]. The first one was indicated for individuals aged 12 years and older, and the second one for children aged 6 months and older, as well as for high-risk adults.

Despite the WHO declaring the end of the pandemic in May 2023, the occurrence of breakthrough infections remains a reality. New strains with additional mutations in the Spike protein (which include the most recent Omicron sublineages (BQ 1.1, BQ1.1, JN.1 [55]) continue to emerge, highlighting the need for ongoing surveillance [6]. To date, there are several approved vaccines against COVID-19 (Table 1), both monovalent and bivalent, with countries adopting different vaccination strategies [56]. Also, more than 350 vaccines are in clinical and pre-clinical development [57]. Despite reported cases and deaths being at reduced levels since the beginning of the pandemic, millions continue to get infected with SARS-CoV-2 variants each month. In this setting, the WHO released, on May 2023, a strategic plan transitioning from emergency status to long-term disease management of COVID-19, with its Strategic Preparedness and Response plan for 2023–2025 [58]. Among the guidelines on disease prevention, control, and management, the plan highlights the importance of anticipating the ongoing changes in the variants (continuing genetic surveillance and collection of real-world data on vaccine effectiveness), evaluating the need for booster dose schedules (especially for different risk groups), and developing new vaccine formulations. Here, it is important to stress the need for effective vaccines able to induce protection not only for healthy individuals but for those with underlying health conditions. Patients with immunosuppression (due to organ transplantation or autoimmune disorders), comorbidities (such as diabetes, chronic respiratory illnesses, cardiovascular diseases), and oncological conditions often exhibit suboptimal immune responses to vaccination [59,60,61] and are at higher risk of severe COVID-19 outcomes. Acknowledging the unique challenges faced by these risk groups is essential for guiding the development of new immunization regimes in order to provide robust and equitable protection. For individuals with comorbidities, for instance, vaccination strategies should address their metabolic dysfunctions and increased baseline inflammation. For those with oncologic conditions or living with HIV, on the other hand, antibody production or cellular responses might be impaired due to ongoing immunosuppressing treatments [37,59,62]. The elderly suffering from age-related immunosenescence might also present suboptimal immune responses to standard vaccine regimens. Here, high-dose vaccination, adjuvanted vaccines, pre-exposure prophylaxis with monoclonal antibodies, or even personalized vaccination schedules are examples of approaches that can be tailored into novel vaccine strategies in order to target these risk groups [62]. Accordingly, booster doses of updated vaccines to the current landscape of SARS-CoV-2 circulating variants are imperative to mitigate COVID-19-related hospital admissions and deaths. These should be encouraged, especially in pre-clinical settings searching for innovative vaccine formulations capable of ensuring broader and long-term protection.

## 3. Next-Generation Vaccines: Where Do We Go from Now?

As new SARS-CoV-2 variants emerge with enhanced transmissibility and ability to evade immunity induced by the existing vaccines, the need for effective alternative strategies for the development of next-generation vaccines has become increasingly urgent. Together with the development of new efficient platforms [63], these strategies could include formulations designed to provide broad protection against a wide range of different coronaviruses, including potential new variants and even mucosal vaccines, offering strong immunity at the primary sites of infection and preventing transmission more effectively. Moreover, the inclusion of conserved epitopes and the association of different adjuvant strategies also comprise other options to improve SARS-CoV-2 control. This topic covers alternative methods to broaden and strengthen immunological memory against SARS-CoV-2.

### 3.1. Mucosal Vaccines and What They Add to the Field

The vaccines currently available to control COVID-19 (which are administered via the intramuscular route (i.m.)) are prone to induce systemic immunity, triggering T cell and serum IgG response. However, they barely stimulate mucosal immune responses, leading to less or no commitment of resident memory T cells, mucosal-homing plasma cells, and secretory IgA (SIgA) production [64]. In this setting, mucosal vaccines bring a promising platform that could enhance immunity in the airways, preventing viral infection, replication, and shedding. Antigens delivered via intranasal (i.n.) or oral routes do reach barrier tissues and non-encapsulated lymphoid follicles known as mucosal-associated lymphoid tissues (MALTs), which are key sites for initiating immune responses. These structures often consist of B cell follicles and follicular dendritic cells, surrounded by T cell areas also containing dendritic cells, macrophages, and plasma cells [65,66]. Within MALTs, specialized microfold cells (M cells) transport antigens from the mucosal lumen to APCs, triggering antigen presentation, T cell priming, and antigen-specific B cell responses [66]. Once activated by APCs in MALTs, CD4^+^ T cells migrate to the B cell follicle to predominantly induce the production of IgA^+^ B cells, which migrate to the mucosal layer. There, they differentiate into plasma cells that secrete IgA, which is later converted into SIgA. This important antibody type protects mucosal surfaces against reinfection by neutralizing pathogens at the entry points. Furthermore, memory T and plasma cells are maintained in MALTs, playing a crucial role in the immune response [67]. Altogether, these features make mucosal vaccines a promising platform to be assessed by future COVID-19 vaccines.

Given the importance of mucosal immunity against COVID-19, researchers have evaluated the potential improvements of applying this technology to approved vaccines. The ChAdOx1-S immunizer, for instance, has been reported to elicit strong Th1 cellular response in rhesus macaques 14 days post i.n. administration, with nAbs, absence of viral pneumonia, and inflammatory disease in the lungs. Notably, no difference was observed in the viral shedding between vaccinated and non-immunized control animals [68]. However, i.n. immunized monkeys showed [69] a significant decrease in SARS-CoV-2 viral load in nasal swabs and lower viral burden in the lungs when compared to control animals. Also, specific IgA and IgG antibodies were detected in the sera from vaccinated animals, as well as in the nasal washes and bronchoalveolar lavages, which were positively correlated with viral control in the respiratory tract. By contrast, a small open-label phase I clinical trial (NCT04816019) did not show evidence of stronger anti-S mucosal IgA and IgG responses upon i.n. vaccination with ChAdOx1-S [70]. It was the first study of COVID-19 in humans showing results from i.n. vaccination, despite the study’s limitations.

Strategies incorporating mucosal vaccines as booster doses have also been assessed. A phase IV open-label clinical trial (ChiCTR2200057278) conducted in China evaluated the effectiveness of different boost strategies in individuals previously immunized i.m. with two doses of inactivated vaccines (CoronaVac or BBIBP-CorV) [71]. Boost strategies included i.m. administration of Ad5-nCoV/Convidecia (CanSino Biologics) and aerosolized Ad5-nCoV orally inhaled through a continuous-vaporing system. The aerosolized booster induced robust mucosal IgA production and durable systemic T cell responses against SARS-CoV-2. Additionally, this strategy also contributed to the longevity of the immune response, as nAbs against the SARS-CoV-2 ancestral strain and Omicron variant were still detected 6 months post-aerosolized vaccination, significantly differing from i.m. vaccination. Another study evaluated an i.n. formulation of trimeric Spike in association with CpG and yielded promising results [72]. Authors reported that K18-hACE2 mice were protected against SARS-CoV-2 challenge when previously immunized with this formulation by i.n. and subcutaneous (s.c.) routes. However, higher effectiveness was observed for the i.n. vaccination strategy when assessing pulmonary clearance of the virus, nAbs titers, and anti-S IgA titers. Also, when the i.n. formulation was used as a heterologous booster in mice previously immunized with ChAdOx1-S i.m. vaccine, the immune response elicited was more robust than the response observed in the homologous ChAdOx1-S immunized control. Another study [73] also demonstrated how mucosal vaccine delivery strategies can impact local immune responses in rhesus macaques. The intratracheal (i.t.) boosting with a bivalent vaccine composed of replication-incompetent human adenovirus type 26 vectors encoding both the ancestral Spike (Ad26.CoV-2.S) and Omicron BA.1 Spike (Ad26.CoV-2.529) elicited robust nAbs and IgA responses in BAL and nasal swabs against the Wuhan, BA.1, BA.5, and BQ.1.1 strains. Furthermore, analysis of bulk-RNA sequencing in BAL showed that vaccination by this route was related to the triggering of Natural Killer (NK) cell activation, APC function, IL-12 signaling, and T and B cell activation. Otherwise, Ad26 i.n. administration induced minimal cellular and humoral mucosal immune responses. Accordingly, almost complete protection against high doses of SARS-CoV-2 BQ.1.1 challenge was observed in the Ad26 i.t. group, whereas the Ad26 i.n. group elicited only partial protection.

Another aspect to be considered is that some studies suggest inhalation delivery might be more efficient in inducing mucosal immunity than the i.n. route [73,74]. This is exemplified by recent reports of mucosal vaccines against SARS-CoV-2 that received emergency use authorization, demonstrating promising effects in stimulating mucosal immunity [65], such as the Ad5-nCoV-IH (Convidecia Air™ in China), which features the same formulation as Ad5-nCoV from CanSinoBio Biologics but reformulated to be aerosolized using a nebulizer and orally delivered [75]. Altogether, the benefits of a needle-free administration strategy are numerous, but several challenges remain to be addressed. By relying upon viral vectors, most mucosal vaccines are susceptible to antivector immune responses, which can impair the development of desired humoral and cellular responses after repeated doses. In that sense, exploring new platforms, adjuvants, and biocompatible polymers (that are not susceptible to host immune responses [76,77]) could help diversify the next generation of mucosal vaccines and bypass this issue.

### 3.2. The Nucleocapsid Protein as a Target of Vaccine Formulations

The Spike protein of SARS-CoV-2 has proven to be an effective target for vaccine development as neutralizing anti-S antibodies elicited by the immunization play a key role in the protection conferred by the first-generation vaccines used to manage the pandemic. However, as it is also a hotspot for mutations, new variants (such as the heavily mutated Omicron) emerge, jeopardizing vaccine-induced immunity. This issue highlights the need for research on more stable viral targets to enhance vaccine efficacy against a broader range of variants. In this regard, the Nucleocapsid (N) from SARS-CoV-2 is a promising target, as it is a largely conserved protein with significant potential for the induction of T-cell cross-reactivity with other betacoronaviruses and emerging variants, making it a good choice for the design of a human pan-coronavirus vaccine. It is a highly immunogenic and abundant protein within the virion, involved in the genome packing during viral replication, and it also plays a role in suppressing the type I IFN pathway [78,79,80]. Previous bioinformatics analyses performed by our group [78] identified robust T and B cell epitopes throughout the N protein, indicating potential targets for vaccine development. Additionally, N-specific cellular memory has been associated with protection against SARS-CoV-2 illness, as children with asymptomatic or mild infections have been reported to exhibit an enhanced CD8^+^ T-cell response to the N antigen [81]. However, the use of an N-based vaccine formulation has proven insufficient to provide protection against SARS-CoV-2 infection. Mathematical models and pre-clinical experiments [79,82,83,84] reported immunization with the Nucleocapsid in vaccine formulations afforded partial or no protection against SARS-CoV-2 disease, despite eliciting Th1-biased adaptive immunity, characterized by both cellular and humoral responses. Furthermore, i.n. administration did not yield improved results [79], as N-based immunization did not induce significant weight loss, viral titers in lung and nasal tissues or pathology amelioration when compared to sham-vaccinated mouse controls.

On the other hand, strategies combining the Nucleocapsid together with the Spike protein [76,82,85] have shown promising results, especially in enhancing the efficacy of existing vaccines. Lam and collaborators (2022) assessed a nasal vaccine formulation consisting of a recombinant Receptor Binding Domain (RBD) portion derived from ancestral or Omicron strains fused with a domain from the Nucleocapsid (referred to as N-RBD). The strategy served as a nasal boost, significantly inducing systemic and mucosa immunity while enhancing neutralizing activity against Omicron in mice previously immunized with mRNA vaccines [86]. Another chimeric protein composed of ancestral RBD and N proteins (referred as SpiN) in association with Poly-ICLC (Hiltonol) adjuvant elicited a robust IFN-γ response from T cells in the spleens and lungs of mice after two i.m. administrations [83]. Central and effector memory T cells were identified as the main source of IFN-γ in the spleens, while tissue-resident memory CD4^+^ and CD8^+^ T cells performed this function in the lungs. Furthermore, IgG responses were detected against both N and RBD domains of the protein. Although no nAbs were detected, SARS-CoV-2 replication was impaired in the animals, leading to preserved lung architecture. This prime-boost strategy effectively controlled Delta and Omicron SARS-CoV-2 VOCs. When evaluated as a booster dose following the administration of commercial vaccines against COVID-19 in humans [87], SpiN demonstrated increased IFN-γ production by CD4^+^ and CD8^+^ T cells, including memory subsets, in individuals pre-vaccinated with CoronaVac, ChadOx1-S, and BNT162b2. This vaccine is currently undergoing a Phase II clinical trial.

Other platforms incorporating chimeric constructions of N and S proteins have been evaluated with promising results. For instance, a lipid-nanoparticle-formulated bivalent vaccine (mRNA S + N), composed of mRNA encoding ancestral N and S proteins (mRNA-N and mRNA-S), elicited stronger S-specific T CD8^+^ response and enhanced neutralizing activity when compared to i.m. mRNA-S immunization in hamsters [84]. Importantly, the augmented response to the S protein, associated with N-specific immunity, conferred faster and more robust lung protection against Delta and Omicron variants. In addition, a combined multi-antigen mucosal approach has also shown promising results [79]. An i.n. vaccine formulation containing trimeric S protein from the Delta variant and N protein adsorbed onto the NanoSTING adjuvant (NanoSTING-SN), a liposome-encapsulated endogenous STING agonist, abolished viral replication in the lungs and nostrils of animals following Delta variant infection. Notably, this vaccine prevented the transmission of the Omicron BA.5 variant in hamsters and offered protection against other sabercoviruses. Furthermore, the immune response in non-human primates was characterized by a long-lasting (6 months) serum IgG response and robust nasal SIgA cross-reactivity against SARS-CoV and MERS-CoV [79]. Taken altogether, preclinical data support the inclusion of N protein in vaccine design strategies aimed to enhance protection against VOCs. Its association with Spike protein has been shown to enhance immunogenicity, resulting in strong and broad protection against SARS-CoV-2 infection.

### 3.3. BCG-Associated Heterologous Immunity, Adjuvant Capacity, and Its Use as Delivery System

A non-conventional approach suggested by many researchers to prevent severe cases of SARS-CoV-2 (especially at the beginning of the pandemic when anti-COVID-19 vaccines were unavailable) was the use of the bacillus Calmette–Guérin (BCG) to induce heterologous immunity [88,89]. This attenuated strain derived from the virulent *Mycobacterium bovis* is the most widely used vaccine in the world, administered to approximately 100 million newborns annually to prevent disseminated tuberculosis (TB) in children [90]. Although BCG is traditionally applied against TB, its long-standing use has provided evidence of non-specific or heterologous effects against unrelated infectious diseases and other inflammatory disorders [91,92]. It has been reported BCG vaccination can protect against viral unrelated respiratory infections, offering variable levels of protection depending on the pathogen and the severity of the infection [93]. In that sense, the innate immune system may play a key role in mediating such effects. Cumulating evidence shows that cross-protection against viral infections is mediated by metabolically and epigenetically reprogramed innate immune cells, including macrophages, neutrophils, and dendritic cells, a feature known as trained immunity. BCG has also been shown to promote heterologous T cell activation [94,95]. Given the heterologous protection observed against several viral infections following BCG immunization, speculations emerged regarding its potential use against SARS-CoV-2 [90,96,97].

This hypothesis was especially strengthened because, during the early phase of the pandemic, observational studies suggested countries with universal BCG immunization programs also seemed to have lower rates of severe COVID-19 cases [89], indicating a possible cross-protection against SARS-CoV-2. However, since these observations were largely epidemiological and influenced by several confounding factors (e.g., differences in healthcare systems, demographics, BCG coverage, etc.), appropriate trials were imperative to draw solid conclusions [98]. Pre-clinical studies aiming to shed some light on the matter investigated BCG immunization in murine models followed by SARS-CoV-2 infection, yielding contrasting findings. While some studies [99,100,101] reported BCG vaccination induced epigenetic alterations in K18-hACE2 mice or Syrian golden hamsters, resulting in reduced viral burden in the lungs to some extent and milder symptoms, others [93,102] reported no significant difference when comparing the BCG-immunized group with the non-immunized group. Given the variations in study design, routes of administration, and BCG strains, these contrasting findings leave the conclusions debatable, suggesting that while BCG vaccination may modulate the immune response, it might not provide robust protection against viral challenges. Worldwide simultaneous clinical trials have also sought to assess whether BCG immunization affects the severity of COVID-19 and mortality rates. These studies took place in countries where neonatal BCG immunization is standard practice and evaluated the outcomes of BCG revaccination in adults. However, the available reports are also heterogeneous and inconclusive [103,104,105], requiring further investigation and leaving the topic without a definitive conclusion.

Despite the different outcomes, the use of the BCG as a trained immunity-based vaccine cannot be dismissed. Its capacity to elicit activation of innate immune cells and to induce heterologous T-cell immunity could be leveraged in vaccine development [95]. This includes the potential for combining BCG with conventional COVID-19 vaccines to enhance protection against the SARS-CoV-2 virus [106]. Along those lines, Perera and collaborators investigated BCG as a priming strategy to enhance i.n. immunization using a human adenovirus (Ad5) expressing full-length S protein from ancestral SARS-CoV-2. When BCG was administered systemically 30 days prior to i.n. vaccination with Ad5-expressing Spike protein, they observed long-lasting protection (up to 6 months post-vaccination) against the Beta variant in K18-hACE2 mice. Furthermore, BCG priming reduced viral shedding, as indicated by the lower viral loads in oral swab samples [106]. Overall, BCG induced non-specific cellular immunity in the lung microenvironment, resulting in the generation of tissue-resident CD4^+^ and CD8^+^ memory T cells. However, no differences in humoral responses against S protein were observed compared to the control. Another promising application of BCG usage is administration alongside SARS-CoV-2 proteins and other adjuvants. Conoupas et al. demonstrated that a single formulation containing BCG, Alum, and Spike from SARS-CoV-2 (termed BCG:CoVac) elicited elevated Th1-biased immune response in K18-hACE2 mice [107]. Furthermore, mice immunized with BCG:CoVac exhibited the highest neutralizing antibody titers when compared to those immunized with Spike with Alum or with BCG with Spike. Notably, mice immunized with BCG:CoVac presented healthy clinical scores, showing no weight loss or detectable viral titers in the lungs.

The design and use of a recombinant BCG (i.e., rBCG) as a vectorizing platform for delivering antigens to the host [108,109,110] also represent an immunization strategy that combines the adjuvant-like properties of the BCG with the ability to induce specific adaptive responses to the vectorized antigens. In this regard, our group previously described [85] a recombinant BCG expressing a chimeric protein derived from SARS-CoV-2 (termed rBCG-ChD6). In the study, we screened both N and S proteins for their most immunogenic peptides and constructed a chimeric protein (rChimera) designed to elicit robust cellular responses (particularly against the conserved Nucleocapsid protein), along with antibodies with neutralizing activity against Spike. We developed a prime-boost strategy consisting of a subcutaneous (s.c.) dose of rBCG-ChD6 followed by a second dose (s.c.) of purified rChimera in combination with Alum. When K18-hACE2 mice were submitted to this immunization regimen and later challenged with SARS-CoV-2, they exhibited significant protection against infection. This was evidenced by high titers of nAbs, strong production of IFN-γ and IL-6 cytokines, reduced viral loads in the lungs, and overall preservation of the lung structure. Other studies have also reported promising results using the rBCG strategy against SARS-CoV-2. For instance, a rBCG expressing the Nucleocapsid protein (rBCG-N) [111] has been reported to induce strong activation of CD4^+^ and CD8^+^ T cells, significant production of IFN-γ, and high titers of anti-Nucleocapsid antibodies. Despite these promising results, the authors did not report any data on SARS-CoV-2 challenge. Another study evaluating a Nucleocapsid-based recombinant BCG [112] reported encouraging findings. In this study, authors assessed the effects of various combinations of formulations containing rBCG-N and recombinant Nucleocapsid or Spike proteins in combination with Alum. Although the study also did not evaluate SARS-CoV-2 challenge, it reported increased neutralizing activity against two VOCs when mice were immunized with rBCG-N + N-RBD-A/N-RBD-A regimen (with the first dose consisting of rBCG-N administered with recombinant N and S proteins with Alum and the second dose comprising recombinant N and S proteins with Alum). Taken together, these studies reinforce the idea that multifaceted approaches can be effective strategies against SARS-CoV-2 and its emerging variants. By integrating various methods, we can enhance the immune response elicited by current and next-generation vaccines, leading to improved control of the virus.

## 4. Future Perspectives

The development and delivery of effective anti-COVID-19 vaccines that are responsive to emerging variants are an urgent priority. The evolving landscape of SARS-CoV-2 variants presents a significant threat, and we must be prepared to prevent breakthrough infections. Next-generation vaccines should aim to provide broader and more durable protection and should also be developed taking into consideration the thermal stability of formulations to ensure vaccines will not need to be stored at ultra-low temperatures (−80 to −60 °C). This would ensure the accessibility of vaccines, especially in resource-limited settings. In that regard, research advancements in lipid nanoparticle formulations aim to enhance the effectiveness of mRNA-based vaccines by protecting the mRNA molecules, improving longer shelf-life, and preventing thermal degradation in transport and storage. Additionally, emerging lyophilization (freeze-drying) techniques offer a promising approach to increasing long-term vaccine stability, preserving the integrity of the components while maintaining immunogenic activity [113,114]. Together, these alternatives are imperative for broader accessibility and effective distribution of vaccines in emergency response scenarios.

All in all, innovative strategies such as those discussed in this review offer promising pathways for enhancing and improving immune responses against SARS-CoV-2 variants. By continuing to adapt and advance vaccine technologies, we can achieve long-term control of COVID-19, paving the way for more effective responses to future pandemics.

## Figures and Tables

**Table 1:** Approved COVID-19 vaccines with WHO EUL/PQ.

Name	Platform	Manufacturer/WHO EUL Holder	Vaccination Schedule/ EUL/PQ Decision Date
Bimervax	Recombinant protein	HIPRA	One dose for individuals 16 years of age and older who have previously received a mRNA COVID-19 vaccine. Intramuscular injection. PQ 9 October 2023.
BNT162b/Comirnaty (ancestral)	Nucleoside modified mRNA	Pfizer/BioNTech	One dose in individuals as of 5 years of age. Two doses with 28 days interval if younger. Intramuscular injection. EUL 31 December 2020.
Original/Omicron (bivalent)			
Omicron/XBB			
Ad26.COV2.S	Recombinant, replication-incompetent Ad26 vectored vaccine encoding the Spike	Janssen/Cilag International NV	One dose for individuals 18 years of age and older. Boosters for adults 18 years of age and older. Intramuscular injection. EUL 12 March 2024.
mRNA-1273/Spikevax	mRNA-based vaccine encapsulated in lipid nanoparticle	Moderna Biotech	Two doses for individuals 6 years of age and older. Intramuscular injection. EUL 30 April 2024.
BBIBP-CorV	Inactivated virus, produced in VERO cells	Sinopharm/Beijing Institute of Biological Products	Two doses for individuals 3 years of age and older, with 28 days interval Intramuscular injection. EUL 07 May 2021.
CoronaVac	Inactivated virus, produced in VERO cells	Sinovac	Two doses for individuals 3–59 years of age, with 4 weeks interval. Intramuscular injection. EUL 1 June 2021.
NVX-CoV2373/Covovax	Recombinant Spike protein formulated with Matrix-M™ adjuvant	Serum Institute of India PVT. LTTD	Individuals 12 years of age and older. Intramuscular injection. EUL 20 December 2021.
NVX-CoV2373/Nuvaxovid: Ancestral	Recombinant Spike protein formulated with Matrix-M™ adjuvant	Novavax	One dose for individuals 12 years of age and older. Intramuscular injection. EUL 20 December 2021.
Nuvaxovid: Omicron XBB1.5			
Ad5-nCoV/Convidecia	Recombinant novel coronavirus vaccine (adenovirus type 5 vector)	CanSino Biologics	One dose for individuals 18–59 years of age. Booster dose for individuals 18 years of age and older. Intramuscular injection. EUL 19 May 2022.
Corbevax	Recombinant RBD from Spike	Biological E	Two doses for individuals 12 years of age and older with 4 weeks interval. Intramuscular injection. EUL 15 January 2024.
ChAdOx1-S/AZD1222/Vaxzevria	Recombinant ChAdOx1 adenoviral vector encoding Spike	AstraZeneca-Oxford	Two doses for individuals 18 years of age and older, with 12 weeks interval Boosters for adults eighteen years of age and older. Intramuscular injection. EUL 16 April 2021. Delisted on 21 March 2024.
Covishield (ChAdOx1_nCoV-19)	Recombinant ChAdOx1 adenoviral vector encoding Spike	Serum Institute of India Pvt. Ltd.a	Two doses for individuals 18 years of age and older, with 12 weeks interval Boosters for adults eighteen years of age and older. Intramuscular injection. EUL 15 February 2021. Delisted on 21 March 2024.
Covaxin (BBV152 A, B, C)	Whole-Virion inactivated Vero cell	Bharat Biotech, ICMR	Two doses for individuals 12 years of age and older. Intramuscular injection. EUL 03 November 2024. suspended. Delisted on 26 April 2024.
SKYCovione (GBP510)	Recombinant protein subunit	SK Bioscience	Individuals 18–65 years of age. Intramuscular injection. EUL 16 June 2023. Delisted on 16 May 2024.

Note: information was gathered from WHO EUL/PQ status of COVID-19 vaccines from 21 May 2024. EUL stands for Emergency Use License and PQ for Prequalification status. Licenses suspended or delisted are also depicted when available.
